# Mosaicism for combined tetrasomy of chromosomes 8 and 18 in a dysmorphic child: A result of failed tetraploidy correction?

**DOI:** 10.1186/1471-2350-10-42

**Published:** 2009-05-18

**Authors:** Gunnar Houge, Helle Lybæk, Sasha Gulati

**Affiliations:** 1 Center for Medical Genetics and Molecular Medicine, Haukeland University Hospital, Bergen, Norway; 2 Department of Clinical Medicine, University of Bergen, Bergan, Norway; 3 Department of Pediatrics, Ålesund Hospital, Ålesund, Norway

## Abstract

**Background:**

Mosaic whole-chromosome tetrasomy has not previously been described as a cause of fetal malformations.

**Case presentation:**

In a markedly dysmorphic child with heart malformations and developmental delay, CGH analysis of newborn blood DNA suggested a 50% dose increase of chromosomes 8 and 18, despite a normal standard karyotype investigation. Subsequent FISH analysis revealed leukocytes with four chromosomes 8 and four chromosomes 18. The child's phenotype had resemblance to both mosaic trisomy 8 and mosaic trisomy 18. The double tetrasomy was caused by mitotic malsegregation of all four chromatids of both chromosome pairs. A possible origin of such an error is incomplete correction of a tetraploid state resulting from failed cytokinesis or mitotic slippage during early embryonic development.

**Conclusion:**

This unique case suggests that embryonic cells may have a mechanism for tetraploidy correction that involves mitotic pairing of homologous chromosomes.

## Background

Unlike meiotic non-disjunctions, mitotic non-disjunctions are rarely observed in humans with the exception of mosaicism for trisomy 8, 9 or 20 [1,2]. In some cases mosaic trisomy of more than one chromosome have been seen [3]. Such mosaic variegated aneuploidy is due to mitotic errors, often associated with premature centromere division [4]. In contrast to mosaic trisomies, the finding of mosaic whole-chromosome tetrasomy is without precedence. Here we present such a patient; a dysmorphic newborn child with mosaicism of leukocytes containing 50 chromosomes due to tetrasomy of chromosomes 8 and 18. This unique clinical case may have relevance concerning the origin of aneuploidy in cancer [5-8] because it indirectly suggests that there might be a mechanism for tetraploidy correction during fetal development that involves mitotic pairing of homologous chromosomes.

## Case presentation

**Figure 1:**
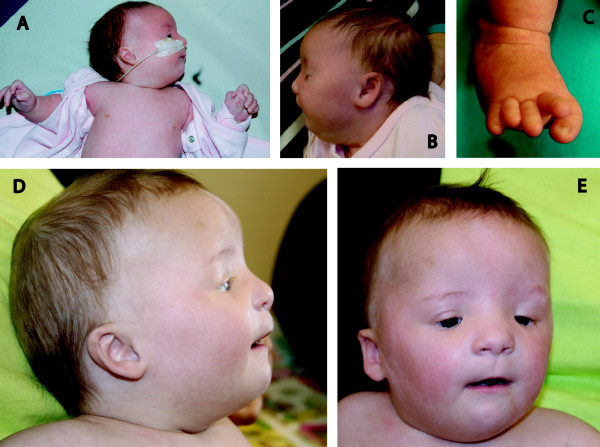
**The child with double tetrasomy 8+18 mosaicism at age 7 weeks (panels A-C) and age 1 year (panels D-E)**.

A baby girl was delivered by cesarean section in week 36 due to maternal hypertension with mild preeclampsia, birth weight 2910 g, length 47 cm. Polyhydramnios was detected at the end of the pregnancy. She had persistent ductus arteriosus (PDA), a small muscular-type ventricle septal defect (VSD) and coarctation of the aorta. The coarctatio aortae was resected at age 6 weeks, and at the same time the PDA was ligated. She has always been short statured: At age 4 months her length was 57 cm (1 cm below 2.5^th ^centile), at age 2 1/2 years 81 cm (4 cm below 2.5^th ^centile). Head circumferences were about 1 cm below the 2.5^th ^centile, e.g. 46 cm at age 2 1/2 years. Major feeding difficulties necessitated gastrostomy at age 4 months. At current age (2 years and 10 months) she still has no interest for food and vomits easily, but feeding her through the enteral feeding tube (Mic-Key^®^) keeps her weight within normal range. On barium-contrast X-ray examination of the esophagus, peristalsis appeared normal without signs of gastro-esophageal reflux. A 24-hour esophageal pH-measurement also gave no indications of reflux. There has been clear psychomotor delay: She started to walk without support at age 2 years and has delayed language development, e.g. at age 2 1/2 years she spoke only 8–10 words but managed quite well by sign language. On neurological examination mirror movements of her hands were found. She also has hearing loss, already suspected before age 2 months and confirmed by brain stem audiometry at age 4 months. On CT-examination of the temporal bone, atretic auditory canals and no middle ear cavities were found. There is marked facial dysmorphism with high frontal hairline, low-set and posteriorly rotated small ears with crumpled helices, inverted epicanthus, short and down-slanting palpebral fissures, no visible eyelashes on lower eyelids, broad nasal root, thin upper lip, small chin and a short neck (Figure 1). A transverse palmar crease was found in the right hand, and on the inside of the left thigh brownish longitudinal streaks were seen. The right foot was deformed with the 1^st ^toe bent in under the 2^nd ^toe (Figure 1). On ophthalmological examination in narcosis, the optic papillae were grayish, and there were some granulations of the retinae in the macular areas. At present, she is an active girl that likes to play. Her only medication is for asthma.

## Results and Discussion

**Figure 2:**
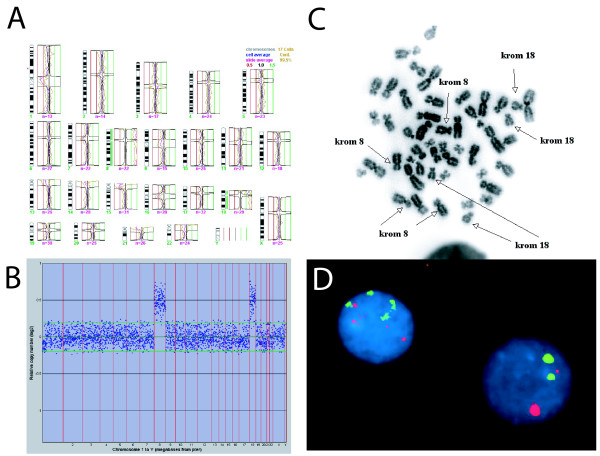
**Chromosome-based high-resolution CGH result (panel A) and 1 Mb BAC-array CGH result (panel B) on a blood DNA sample collected at age two days**. The apparent 50% increase in DNA amount corresponding to chromosomes 8 and 18 was due to double tetrasomy of both chromosomes (panel C, showing a double tetrasomic metaphase). By interphase FISH, four copies of chromosomes 8 and 18 were detected in 15% of the cells from a 3-days PHA-stimulated blood culture (panel D).

Because the girl was dysmorphic with major feeding difficulties, blood samples were drawn two days after birth for chromosome investigations; routine G-banding and chromosome-based high-resolution comparative genomic hybridization (HR-CGH). The G-banded karyotype, based on screening of ten metaphases from a phytohemagglutinin-stimulated 3-day blood lymphocyte cultures, was normal. Surprisingly, the HR-CGH result that came a few weeks later suggested a combination of non-mosaic trisomy 8 and trisomy 18 (Figure 2, panel A). An identical finding was subsequently done on a 3500 BAC-clone array-CGH platform made by the Norwegian Microarray Consortium (for details, see [9]), the ratio still indicating a 50% increase in DNA amount corresponding to chromosomes 8 and 18 (Figure 2, panel B). To investigate if uniparental disomy of other chromosomes might be present, or if abnormal copy number variants below the resolution limit of the 3500 BAC-clone array might be found, we have recently examined DNA from the child's original blood sample (taken at age 2 days) on the Affymetrix Genome-Wide Human SNP Assay Kit version 6.0 (Affymetrix, Inc., CA). No indication of uniparental disomy (i.e. larger regions of homozygocity) was found, and no additional (and structural) de novo copy number abnormalities were detected.

A re-examination of the original leukocyte cell culture suspension with FISH-probes for centromeres 8 and 18 explained the surprising CGH finding: In 2% of the metaphases (100 metaphases examined) and 15% of the interphases (200 nuclei examined) tetrasomy for both chromosomes were found (Figure 2, panels C and D). The discrepancy between the CGH-findings (50% dose increase for 8 and 18, suggesting that 50% of the cells had four chromosomes 8 and 18) and the cell culture findings (15% of the interphases had four chromosomes 8 and 18) were likely due to negative selection of double tetrasomic cells during PHA-stimulated culturing of blood leukocytes. This illustrates that conventional G-banded karyotyping may easily overlook such mosaicism. To investigate other tissues for similar mosaicism, a skin biopsy from the inside of the left thigh was collected for fibroblast culturing at age 4 months, and at age 14 months a buccal smear was collected. In none of these tissues double tetrasomic cells were found by interphase centromere 8+18 FISH with 200 nuclei examined in each case. At age 20 months, a new blood sample was taken, and the double tetrasomic cells could no longer be detected, neither by CGH nor by interphase FISH.

To determine the origin of the four extra chromosomes (two chromosomes 8 and two chromosomes 18) that probably were present in around 50% of the blood leukocytes at the time of birth, microsatellite markers for chromosomes 8 and 18 were compared between blood-DNA samples from the parents and the original child blood-DNA sample taken at age 2 days (Table 1). There was no indication of more than two alleles for any simple tandem repeat examined, making meiotic non-disjunction an unlikely mechanism. Furthermore, there was no systematic skewing of the ratios between maternal and paternal allele peak sizes (Table 1), which would have been the case if three of the chromosomes in each quadruple were uniparental. Taken together, this indicates that the mosaicism was a consequence of mitotic events, and that the origin is a mitotic division where all four chromatids of two chromosome pairs (8 and 18) segregated to one daughter cell only.

**Table 1:** Allele sizes of polymorphic chromosome 8 and18 simple tandem repeats.

**UniSTS**	**Position (Mb from pter)**	**Mother (m)**	**Father (p)**	**Child**	**Ratio m/p peak heights**
**Chrom. 8:**					
D8S264	21	153–155	143–153	143–155	0,50
D8S1104	41	135–135	143-143	135–143	1,01
D8S268	41	260–266	264–266	260–264	1,24
*D8S531*	49	121–127	123-123	121–123	1,60
*G08718*	49	219-219	214-214	214–219	0,94
D8S517	53	251–253	255–258	251–255	1,37
D8S260	62	207–215	209–215	207–215	1,05
D8S277	65	165–173	165–169	169–173	1,18
D8S270	93	101–112	110–112	101–110	1,84
D8S1784	106	288-288	282–286	282–288	0,71
D8S550	109	194–212	210-210	210–212	0,65
					
					**Mean 1,03**
**Chrom. 18:**					
D18S452	6	128–144	132–136	136–144	1,00
D18S53	11	165–173	165–169	169–173	1,19
D18S453	13	148–152	152-152	148–152	2,13
D18S71	13	270–278	258–276	258–278	0,55
D18S73	13	142–144	142–146	144–146	1,98
*D18S1104*	17	148–152	141–148	141–148	1,10
*D18S1149*	17	258–265	263–267	263–265	0,77
*D18S869*	18	186–198	186–189	186–198	1,39
D18S478	23	248–250	246-246	246–250	1,21
D18S1102	33	90–94	90–92	90–94	0,97
D18S474	47	124–126	132–138	126–138	2,68
D18S61	66	228–230	232-232	228–232	2,13
D18S1161	70	231–233	219-219	219–233	0,79
					
					**Mean 1,24**

The ratio between the maternal and paternal peak heights is given in the right column, with the geometrical means of each chromosome in bold. The names of the centromeric repeats are written in italics.

The finding of tetrasomy for two autosomes in a newborn is without precedence. To the best of our knowledge, even single chromosome tetrasomy mosaicism has not previously been reported – except in cancer cells. The reason for the uniqueness of our finding is either that this indeed is a very rare chromosomal aberration, or that this type of malsegregation commonly occurs but is not detected due to negative selection against aneuploid cells during embryonic development and the culturing of blood cells for routine karyotyping. We were fortunate to observe this because we performed "unbiased" CGH analyses on leukocyte DNA from a newborn. Moreover, extra copies of both of the involved chromosomes are known to be compatible with sustained cell growth in the embryo, increasing the likelihood that a double tetrasomic cell line could survive to term. Notably, the initial cytogenetic investigation (G-banding) appeared normal – only a later reexamination by FISH revealed double tetrasomic cells and metaphases (Figure 2). Apparently, the short-term PHA-stimulated leukocyte culture decreased the number of aberrant cells from around 50% to 15%. Twenty months later the aberrant clone was undetectable in blood, probably because it was counter-selected in the bone marrow.

At a later time point we were unsuccessful in finding double tetrasomic cells in other tissues, i.e. in squamous epithelial cells from a buccal smear and cultivated fibroblasts from a skin biopsy taken from left groin, where skin pigment mosaicism could be seen. The reason for this, at least when the fibroblasts are concerned, can be the unpredictable distribution of mosaicism and not necessarily a negative selection process [10]. Even though we lack cytogenetic proof that the mosaicism affects other tissues than the bone marrow, the clinical picture suggests this. The child's phenotype has elements of resemblance to children with mosaicism for trisomy 8 or trisomy 18 (Table 2). In fact, there are no known physical features in the patient, with the possible exception of poorly developed lower eyelashes, that has not been reported in patients with trisomy 8 or 18 mosaicism [11-13].

**Table 2:** The patient's phenotypic features compared to cases with mosaic trisomy 8 or 18

**Our patient: Double tetrasomy 8+18 mosaicism**	**Trisomy 8** **mosaicism**	**Trisomy 18** **mosaicism**
Short stature		**+**
Small head	**+**	**+**
Feeding problems		**+**
Developmental delay	**+**	**+**
Deafness, conductive	**+**	
High frontal hairline/prominent forehead	**+**	**+**
Low-set/posteriorly rotated ears	**+**	**+**
Crumpled ear helices		**+**
Narrow/atretic auditory canals	**+**	
Middle ear abnormalities		**+**
Short palpebral fissures		**+**
Epicantic folds		**+**
Downslant	**+**	
Broad nasal bridge	**+**	**+**
Thin upper lip		**+**
Small chin	**+**	**+**
Short neck	**+**	**+**
Skin pigmentation anomalies	**+**	
Overriding toes		**+**
Coarctatio aortae		**+**
Ventricular septal defect (VSD)	**+**	**+**
Persistent ductus arteriosus (PDA)	**+**	**+**
Retinitis pigment.-like findings in retina	**+**	

This case is of particular interest because it illuminates early events in embryogenesis that may have implications for tumor biology. Our data suggests that all four chromatids of the homologous pair were pulled to one daughter cell only by the mitotic spindle apparatus, analogous to the syntelic attachment of the mitotic spindle that may be seen in tetraploid yeast cells [5]. Unlike other organisms, pairing of homologous chromosomes in somatic cells is commonly seen in Dipterians such as *Drosophila *and mosquitoes [14]. Notably, homologous pairing in both meiosis and mitosis occurs independently of synapsis and recombination [14]. Furthermore, the finding of (mosaic) segmental isodisomy as a disease mechanism in some cases of imprinting-related growth syndromes (e.g. Beckwith-Wiedemann syndrome and Russell-Silver syndrome, [15]) also indicates that mitotic homologous pairing takes place. That such pairing is not limited to imprinted chromosomes are illustrated by the reports of children with recessive diseases due to homozygocity for mutations carried by one of the parents only, the disease manifesting due to segmental isodisomy formation [16-19].

We believe that the most likely explanation for the double tetrasomy is that the starting point was a tetraploid state, which is the normal situation after S-phase, or a tetraploid cell line. If the origin was a tetraploid state, aborted S-phase (mitotic slippage) or interrupted M-phase (failed cytokinesis) are possible mechanisms. Chromatid non-disjunction is one suggested reason for failed cytokinesis [6,8]. If the origin was a tetraploid cell line, there are scant indications that such cells may later become diploid. In *Candida albicans *tetraploid strains become diploid or near diploid through "concerted chromosome loss" [20]. In hepatic cells being tetraploid after fusion to bone marrow stem cells, a "reduction mitosis" appears to be able to transform tetraploid hybrids into diploids [21]. In both cases the mechanism is unknown. A variable number of tetraploid cells is commonly found in chorion villus or amniocyte cultures, but such cells are rarely found in liveborns [22]. The origin can be meiotic or mitotic errors [23,24].

**Figure 3:**
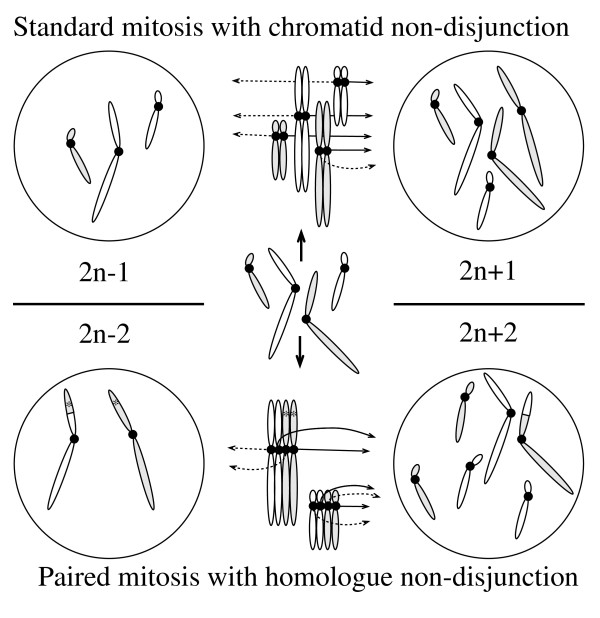
**A drawing that exemplifies the hypothesized mitotic homologue non-disjunction**. The upper part of the figure shows two separate homologue pairs from a conventional mitosis, and in one chromosome a chromatid non-disjunction occurs. The lower part of the figure illustrates two pairs of homologues from a mitosis where pairing has occurred between all homologue chromosomes, and where all (four) chromatids of one such pair segregates to the same daughter cell. We suggest that this might have happened very early in development to two homologue pairs, made by chromosomes 8 and 18. We also illustrate how a detrimental mutation that has arisen during replication (marked by an asterix) may be eliminated by segregation to one daughter cell only after a mitotic cross-over that also generates a segmental (and terminal) uniparental isodisomy.

Conceivably, failed cytokinesis or mitotic slippage might be quite common events in early embryogenesis, for instance during the rapid cell cycles taking place in the peri-gastrulation stage of mammalian embryogenesis [25]. If there exists a special mechanism to deal with this, e.g. to reinitiate the spindle apparatus after the failure has been corrected, balanced segregation would be an advantage, and this could require pairing of the homologues. A further hypothetical advantage of such pairing is removal of replication errors or detrimental mutations from at least one of the daughter cells by segregating both chromatids of one homologous pair to the same daughter cell, if necessary after mitotic crossovers. In the latter case uniparental isodisomy (UPD) for the whole or a segment of a chromosome would be the result. Our hypothesis is illustrated in Figure 3, modeling single chromosome aneuploidy due to chromatid non-disjunction and double chromosome aneuploidy caused by paired homologues non-disjunction.

## Conclusion

This unique case indirectly suggests that a mechanism for tetraploidy correction involving pairing of homologues may be present in somatic cells, and that mosaicism originating in tetraploidization could be a cause of developmental abnormalities that usually remain undetected. When such a mechanism is dysfunctional, aneuploidy is a likely result – which is commonly found in many types of cancer.

## Consent

The parents have given written consent to publication of the patient's pictures, and the parents have also seen and approved the publication of the disease history.

## Competing interests

The authors declare that they have no competing interests.

## Authors' contributions

GH initiated the study, and was the person mainly responsible for its design and conclusions. HL carried out the molecular genetic studies and participated in the writing of the manuscript. SG collected clinical data and participated in the writing of the manuscript. All authors have read and approved the final manuscript.

## Pre-publication history

The pre-publication history for this paper can be accessed here:


